# Serological Evidence of Influenza A Virus Exposure and Reactivity to an H1N1pdm09 Reference Strain in Backyard Pigs in Chile

**DOI:** 10.3390/vetsci13101109

**Published:** 2026-10-17

**Authors:** Galia Ramirez-Toloza, Naomi Ariyama, Constanza Urzua, Raúl Alegría, Benjamín Bennett, Patricio Pérez, Catalina Pardo-Roa, Rafael Medina, Víctor Neira

**Affiliations:** 1 Facultad de Ciencias Veterinarias y Pecuarias, Universidad de Chile, Santiago 8820808, Chile; galiaram@uchile.cl (G.R.-T.); constanza.urzua.e@uchile.cl (C.U.); benjamin.bennett@ug.uchile.cl (B.B.); 2 Facultad de Medicina Veterinaria y Agronomía, Universidad de Las Américas, Santiago 7500975, Chile; nariyama@udla.cl; 3 Escuela de Medicina Veterinaria, Facultad de Medicina, Facultad de Ciencias Biológicas y Facultad de Agronomía y Sistemas Naturales, Pontificia Universidad Católica de Chile, Santiago 7820436, Chile; raul.alegria@uc.cl; 4 Servicio Agrícola y Ganadero (SAG), Santiago 8320000, Chile; patricio.perez@sag.gob.cl; 5 Department of Child and Adolescent Health, School of Nursing, Pontificia Universidad Católica de Chile, Santiago 7820436, Chile; cpardo1@uc.cl (C.P.-R.); rafael.medina@emory.edu (R.M.); 6 Departamento de Enfermedades Infecciosas e Inmunología Pediátrica, Escuela de Medicina, Pontificia Universidad Católica de Chile, Santiago 8330024, Chile; 7 Department of Microbiology, Icahn School of Medicine at Mount Sinai, New York City, NY 10029, USA; 8 Department of Pathology and Laboratory Medicine, School of Medicine, Emory Vaccine Center, Emory University, Atlanta, GA 30329, USA

**Keywords:** influenza A virus, backyard pigs, serology, H1N1pdm09, hemagglutination inhibition, Chile

## Abstract

This study assessed serological exposure to influenza A virus (IAV) in swine backyard production systems (BPS) across 14 regions of Chile. Sera originated from official surveillance for exotic swine diseases in 2020. Database reconciliation identified 928 sample records linked to 123 distinct registered premises; two empty tubes were excluded from sample-level analyses. Nineteen of 926 evaluable samples were ELISA-positive (2.05%; descriptive 95% confidence interval [CI]: 1.32–3.18%), from six premises (4.88%; 95% CI: 2.25–10.23%). Seven initially doubtful samples were retested, yielding six positive and one negative result. Thirteen of the 19 ELISA-positive samples had sufficient residual serum for hemagglutination inhibition (HI) testing; all 13 reacted to the H1N1pdm09 reference strain (titers, 1/40–1/640). Positive premises were located in O’Higgins, Biobío, and Ñuble. The results support IAV exposure and HI reactivity to an H1N1pdm09 reference strain, but do not identify the infecting genotype, active circulation, or direction of transmission. Surveillance-based selection and within-premises clustering limit population-level inference.

## Simple Summary

We assessed influenza A virus exposure in backyard pigs using sera from official surveillance across 14 regions of Chile in 2020. Nineteen of 926 evaluable samples were ELISA-positive, from six of 123 registered premises. Thirteen positive samples had sufficient serum for hemagglutination inhibition testing, and all reacted to a Chilean H1N1pdm09 reference strain. The results support previous exposure to antigenically related influenza A viruses but do not identify humans or other pigs as the source of infection. These findings support continued surveillance and biosecurity in backyard production systems.

## 1. Introduction

Influenza A viruses (IAVs) are single-stranded, negative-sense RNA viruses with linear genomes divided into eight segments. They belong to the genus Alphainfluenzavirus in the family Orthomyxoviridae [1]. IAVs are common pathogens of horses, swine, humans, and domestic poultry; they also cause sporadic or geographically limited infections and disease in other mammals [2]. In swine, influenza A is characterized by a highly contagious respiratory disease with high morbidity and low mortality, which can reduce production performance on intensive swine farms [3]. From a public health perspective, both human-to-pig and pig-to-human transmission have been described [4,5]. Since the 2009 H1N1 pandemic, active surveillance programs have been established worldwide to identify emerging reassortant IAVs [2]. In addition, swine are considered intermediate hosts for IAV transmission between birds and humans, particularly in areas where poultry and swine have frequent contact [6]. As in most parts of the world, intensive poultry and swine farms in Chile are separated. However, in small-scale backyard production systems (BPS), farmers often keep diverse bird species, including chickens, ducks, geese, turkeys, guinea fowl, and quail, alongside mammals such as pigs, goats, cattle, horses, and rabbits, while biosecurity practices are limited [7,8]. Consequently, reassortment and the emergence of novel IAVs in backyard swine may occur. Although IAV has been reported in backyard swine populations in Chile [9,10,11], previous studies were restricted to three administrative regions near the capital and included only a limited number of samples. In this study, we comprehensively assessed IAV seropositivity in swine BPS across a broad geographic area comprising 14 of Chile’s 16 administrative regions.

## 2. Materials and Methods

Samples originated from Servicio Agrícola y Ganadero (SAG) surveillance for exotic swine diseases, with reception dates between 17 January and 25 November 2020. The surveillance program sought broad coverage of known swine BPS, and this study used the available sera from 14 regions, from Arica and Parinacota to Los Lagos. This was not a probability sample designed specifically for IAV. There were 143 linked submission protocols, each containing at most 10 records; some premises contributed multiple submissions. The field sampling rule was a maximum of 10 samples per premises per sampling occasion. The database records reception dates rather than sampling dates: 11 premises–reception-date groups contained 12–20 records across multiple protocols, so these dates cannot independently verify distinct sampling occasions. The available records do not establish whether repeated submissions always represented different animals. Weaned or adult pigs were sampled. Whole-blood samples were sent to the Laboratorio Virología Animal, SAG, Lo Aguirre, where serum was separated by centrifugation. Aliquots were submitted to the Animal Virology Lab, Universidad de Chile. The source laboratory database contained 954 sample records. Twenty-six records from three submission protocols were excluded because they could not be linked to the required premises metadata in the available GIS/BD sheets. These records had negative ELISA results and are retained in the Supplementary Dataset with an exclusion flag. These 26 records were excluded from all analyses to maintain a uniform analytical process. The remaining 928 records were linked to 123 distinct official premises identifiers. Two records represented empty tubes, leaving 926 evaluable ELISA results. All 123 registered premises had at least one evaluable sample.

Antibodies against IAV nucleoprotein (NP) were detected by enzyme-linked immunosorbent assay (ELISA) using the ID Screen^®^ Influenza A Antibody Competition Multi-Species kit (IDvet, Grabels, France) [12]. Samples were initially tested in single wells following the porcine-serum procedure in the supplied manufacturer’s insert (FLUACA ver 0917 ES). Each sample well contained 5 µL of serum and 200 µL of Diluent 2. Kit-supplied positive and negative controls were tested in duplicate, using 10 µL of control and 90 µL of Diluent 2 per well. Incubation, washing, anti-NP–HRP conjugate, and TMB development followed the manufacturer’s instructions; OD was read at 450 nm. The supplied wash buffer and 0.5 M stop solution were used. Their complete chemical composition and the acid identity of the stop solution are not specified in the insert. Results were expressed as S/N% = (ODsample/mean ODnegative control) × 100. S/N% ≤ 45 was positive, 45 < S/N% < 50 was doubtful, and S/N% ≥ 50 was negative. Initially doubtful samples were retested, and repeat results determined their final classification. The assay measures anti-NP antibody competition without separately quantifying immunoglobulin isotypes.

ELISA-positive samples with sufficient residual serum were tested by hemagglutination inhibition (HI) using the Chilean H1N1pdm09 reference strain A/swine/Chile/VN1401-559/2014(H1N1). Given the limited serum volumes available, this previously characterized strain was selected as a reference antigen based on its published genetic and antigenic characterization [13,14,15]. In that characterization, antiserum to strain 559 showed broad cross-reactivity within the tested Chilean A(H1N1)pdm09-like antigenic cluster, supporting its use as a reference antigen. This rationale does not establish that the assay detects all viruses circulating in 2020 or that the strain represents every virus to which the pigs may have been exposed.

The HI assay was performed according to Kitikoon et al. (2014), using turkey erythrocytes at a working concentration of 0.5% [16]. Serum pretreatment included heat inactivation, kaolin treatment, and erythrocyte adsorption to remove nonspecific inhibitors and hemagglutinins. Quality-control procedures included erythrocyte controls to verify appropriate settling, serum controls without virus to detect nonspecific hemagglutination, homologous positive-control antiserum, and antigen back-titration to verify the hemagglutinating activity used in each assay [16]. HI titers were expressed as reciprocal serum dilutions, with ≥40 considered positive. The study characterized HI reactivity among ELISA-positive sera and was not designed to compare the sensitivity of the two assays.

Premises were georeferenced using Servicio Agrícola y Ganadero (SAG) records. Spatial analysis was performed using SaTScan version 10.3 and Kulldorff’s spatial scan statistic with a Bernoulli model in a purely spatial analysis [17,18,19]. The unit of analysis was the registered swine premises (BPS). Premises with confirmed positive results were defined as cases, whereas premises with confirmed negative results were defined as controls. Repeated submissions were not treated as independent observations, and only one result per premises was included in the analysis. Samples with doubtful results were retested to establish their final status, and no premises with unresolved doubtful results were included. A circular spatial scanning window was used, with the maximum spatial cluster size set to the default of 50% of the population at risk. Statistical significance was assessed using 999 Monte Carlo replications. The analysis included 123 registered premises. The spatial cluster comprised 41 premises, including four positive premises (Farms 2–5). Maps were prepared using QGIS [20] and SaTScan [21]. The analysis assessed the spatial distribution of seroreactivity rather than transmission rates. The small number of positive premises, together with the surveillance-based sampling design and the potential influence of the underlying geographic distribution of registered premises, limits the interpretation of the observed spatial clustering.

Sample positivity was calculated among records with a final ELISA result. A registered premises was positive if at least one of its samples was positive. Descriptive 95% Wilson confidence intervals were calculated for sample and premises proportions, overall and by region. These intervals were not adjusted for within-premises clustering, selection probabilities, or diagnostic sensitivity and specificity.

## 3. Results

The final dataset contained 926 evaluable ELISA results from 928 records within the documented study frame. Sample-level and registered-premises-level positivity are reported separately in Table 1. One empty tube from Antofagasta and one from Coquimbo were excluded from sample-level denominators.

**Figure 1:**
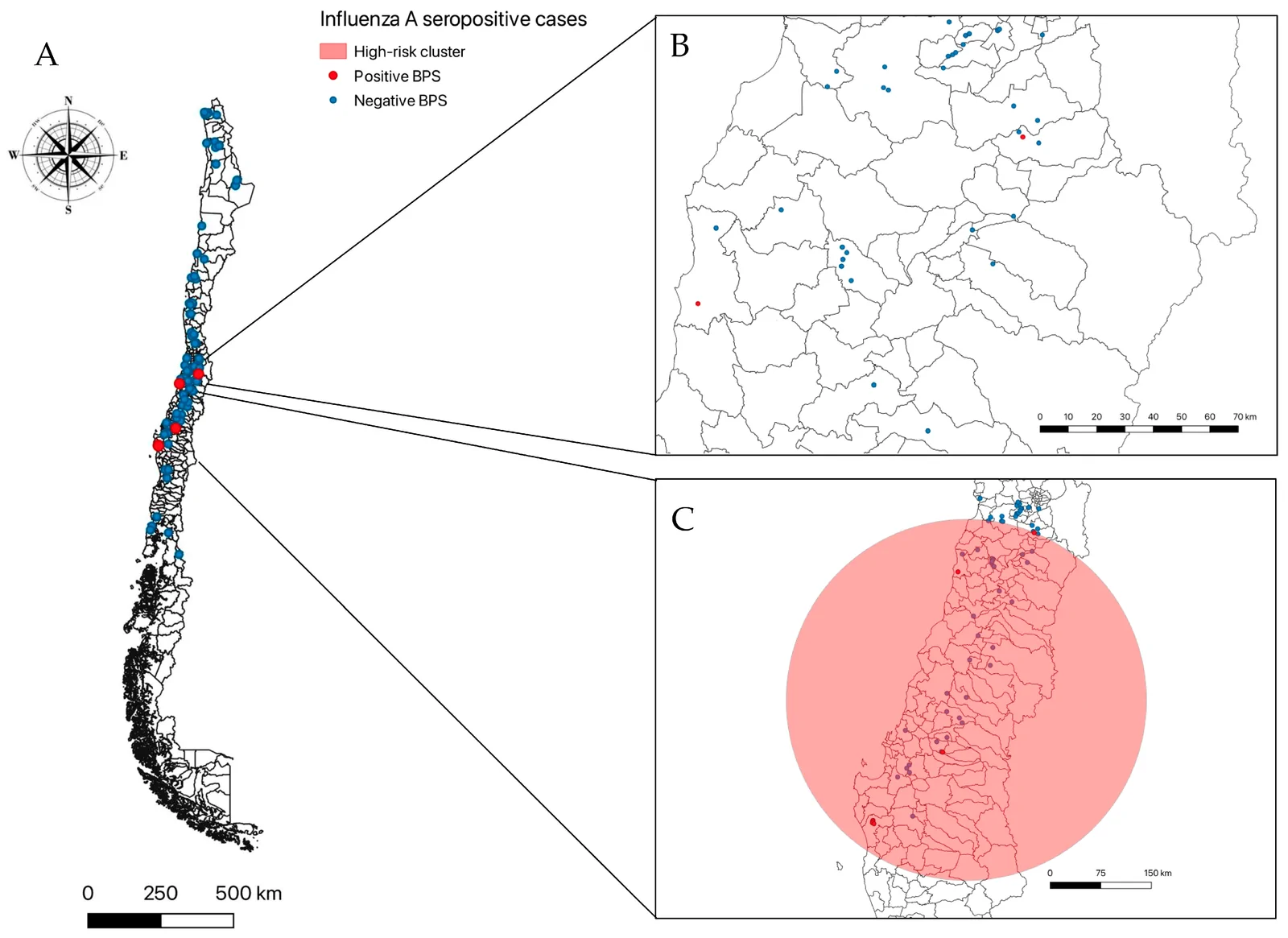
Spatial distribution of sampled swine backyard production systems (BPS) across 14 administrative regions of Chile based on final ELISA classification. (**A**) National overview of sampled BPS; (**B**,**C**) are insets of the areas outlined in (**A**); the shaded circle in (**C**) displays the high-risk spatial cluster (radius = 266.40 km, *p* = 0.046, RR = 17.22). Blue symbols indicate final negative premises; red symbols indicate final positive premises (*n* = 6). All initially doubtful samples were resolved upon repeat testing. Exact premises coordinates and coordinate grids are withheld to protect holding confidentiality.

**Figure 2:**
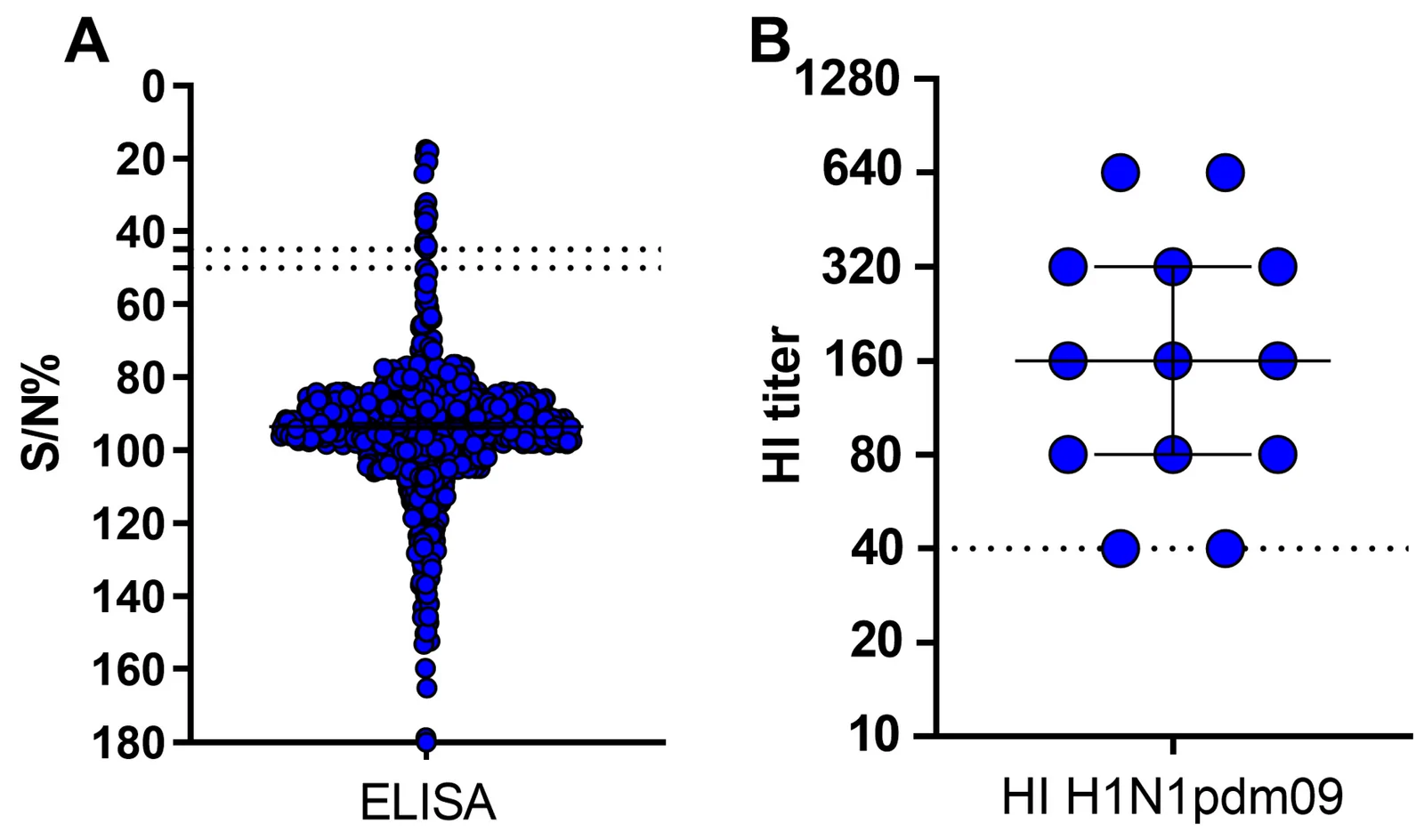
ELISA and hemagglutination inhibition (HI) results. (**A**) Final ELISA results. Horizontal dotted lines indicate the positive cutoff (S/N ≤ 45%) and negative cutoff (S/N ≥ 50%), defining the doubtful range (45% < S/N < 50%). (**B**) Reciprocal HI titers for the 13 ELISA-positive samples tested against the Chilean H1N1pdm09 reference strain A/swine/Chile/VN1401-559/2014(H1N1). The horizontal dotted line denotes the positivity threshold (reciprocal titer ≥ 40).

Nineteen of 926 evaluable samples were ELISA-positive (2.05%; 95% CI: 1.32–3.18%). Thirteen were initially positive. Seven additional samples were initially doubtful; retesting classified six as positive and one as negative, leaving no persistently doubtful samples. Six of 123 registered premises were positive (4.88%; 95% CI: 2.25–10.23%): three in Biobío, two in O’Higgins, and one in Ñuble. Within-positive-premises sample positivity ranged from 20% to 80%, and three premises had ≥50% positive samples. HI reactivity supported the presence of antibodies cross-reactive with the H1N1pdm09 reference strain. Thirteen of the 19 ELISA-positive samples had serum for HI testing, and all of them reacted to the H1N1pdm09 reference strain, with titers of 1/40–1/640. The six samples that became positive after ELISA retesting had insufficient residual serum for HI. HI results represented four of the six positive premises; Farms 2 and 3 had no HI results. Positive premises are designated Farms 1–6 to protect their identities (Figure 1 and Figure 2).

Farm 1 was described in the original geographical assessment as being within 800 m of a commercial swine farm. No formal analysis of the association between proximity and seropositivity was performed. Farms 2, 3, and 4 were located in the same county in southern Chile. Farm 4 had the highest within-premises positivity (4/5; 80%). Farm 6, an educational farm, had six positive samples (6/10; 60%) and the highest HI titers (160–640) (Figure 2B). Farm 5 contributed two submission protocols, totaling 20 samples, including five positives.

The spatial analysis reported a cluster centered at a location withheld to protect premises confidentiality, with a radius of 266.40 km (*p* = 0.046; RR = 17.22; Figure 1C). These estimates have been independently recalculated following data reconciliation. The cluster includes Farms 2–5 (Figure 1C).

## 4. Discussion

The results provide serological evidence of IAV exposure in backyard pigs across a broad geographical area of Chile. All 13 sera tested by HI reacted to the H1N1pdm09 reference strain, supporting exposure to antigenically related viruses (Figure 2B). Tapia et al. [14] identified distinct Chilean H1 antigenic clusters, with no cross-reactivity between the tested clusters, and broad within-cluster reactivity of antiserum to strain 559. Antigenically distant A(H1N1)pdm09-like variants were also observed. This characterization supports the reference-antigen choice but does not establish uniform sensitivity to all viruses circulating in 2020. HI reactivity does not identify the neuraminidase subtype or the complete viral genotype; H1N2 and H3N2 are subtypes rather than antigenic lineages.

The geographical grouping of positive premises may help guide future surveillance, but the original spatial result requires cautious interpretation. Only six premises were positive, and surveillance coverage and the underlying density of BPS may influence the observed pattern. Movement of pigs between premises is a possible source of exposure [9], but transmission links were not investigated.

Farm 6 warrants discussion because it was an educational farm with six ELISA-positive samples and HI titers of 160–640 among the five sera tested. Frequent human–animal contact makes reverse zoonotic exposure a plausible hypothesis. However, these results cannot distinguish human-to-pig transmission from introduction through infected pigs or subsequent pig-to-pig transmission. Human–swine transmission is relevant to IAV ecology [4,5,22,23,24], supporting attention to biosecurity in educational and backyard settings without identifying a specific transmission event.

Only one positive BPS was described as being near a commercial swine farm. This observation does not demonstrate absence of an association with proximity or exclude transmission between production systems. No formal proximity analysis was performed. Previous investigations of porcine reproductive and respiratory syndrome in Chile illustrate the importance of biosecurity and connections between backyard and commercial systems [25].

Previous studies in central Chile reported IAV seropositivity in BPS [9,11]. Jimenez-Bluhm et al. (2018) reported ELISA seropositivity of 2.4% among pigs sampled in O’Higgins [11]. Another study reported swine seroprevalence of 6.3% and pooled-sample RT-PCR positivity of 2.6% in Valparaíso, Metropolitan, and O’Higgins [9]. Our study extends the geographical coverage of serological surveillance and provides HI results for 13 ELISA-positive sera. The source and direction of transmission remain unresolved. Reverse zoonotic transmission is one possible explanation [23], alongside transmission between pigs.

Several limitations should be considered. Available surveillance sera were analyzed, so the estimates describe the tested samples and registered premises rather than nationally representative prevalence. Sample-level confidence intervals are unadjusted for clustering within premises and may understate uncertainty. Multiple submissions and incomplete individual-animal identification limit inference about independent animals. Serology cannot determine current shedding, infection timing, the infecting genotype, or transmission direction. HI used a single reference antigen and was restricted to ELISA-positive samples; responses to antigenically divergent viruses and HI reactivity among ELISA-negative samples were not assessed. Six positive sera could not be tested by HI, and two positive premises had no HI characterization. Although antibodies can persist beyond the period of detectable virus, direct virological surveillance is needed to characterize circulating viruses [26].

## 5. Conclusions

IAV antibodies were detected in 19 of 926 evaluable surveillance samples from backyard pigs in Chile. All 13 sera tested by HI reacted to the H1N1pdm09 reference strain. These findings support previous exposure and antigenic relatedness to the test strain, while leaving the viral genotype and transmission source unresolved. Continued serological and virological surveillance is warranted across backyard production systems, including educational farms.

## Figures and Tables

**Table 1:** Final IAV ELISA results by region, with descriptive 95% confidence intervals (CI).

Region	Tested Samples	Positive Samples	Sample % (95% CI)	Positive/ Tested BPS	BPS % (95% CI)
Arica y Parinacota	89	0	0.00 (0.00–4.14)	0/9	0.00 (0.00–29.91)
Tarapacá	70	0	0.00 (0.00–5.20)	0/7	0.00 (0.00–35.43)
Antofagasta	24	0	0.00 (0.00–13.80)	0/7	0.00 (0.00–35.43)
Atacama	38	0	0.00 (0.00–9.18)	0/7	0.00 (0.00–35.43)
Coquimbo	37	0	0.00 (0.00–9.41)	0/11	0.00 (0.00–25.88)
Valparaíso	60	0	0.00 (0.00–6.02)	0/7	0.00 (0.00–35.43)
O’Higgins	147	7	4.76 (2.33–9.50)	2/16	12.50 (3.50–36.02)
Maule	85	0	0.00 (0.00–4.32)	0/8	0.00 (0.00–32.44)
Ñuble	87	5	5.75 (2.48–12.76)	1/7	14.29 (2.57–51.31)
Biobío	61	7	11.48 (5.67–21.84)	3/11	27.27 (9.75–56.56)
La Araucanía	34	0	0.00 (0.00–10.15)	0/5	0.00 (0.00–43.45)
Los Ríos	7	0	0.00 (0.00–35.43)	0/1	0.00 (0.00–79.35)
Los Lagos	35	0	0.00 (0.00–9.89)	0/5	0.00 (0.00–43.45)
Metropolitan	152	0	0.00 (0.00–2.46)	0/22	0.00 (0.00–14.87)
Total	926	19	2.05 (1.32–3.18)	6/123	4.88 (2.25–10.23)

One empty tube in Antofagasta and one in Coquimbo were excluded. Initially doubtful samples were from O’Higgins (2; one repeat positive, one negative), Biobío (4; all repeat positive), and Ñuble (1; repeat positive). No samples remained doubtful. Premises counts use distinct official identifiers. Confidence intervals are descriptive and unadjusted for clustered sampling.

## Data Availability

The original contributions presented in this study are included in the article/Supplementary Material. Further inquiries can be directed to the corresponding author.

## Supplementary Materials

The following supporting information can be downloaded at https://www.mdpi.com/article/10.3390/vetsci13101109/s1. Supplementary Dataset S1 contains pseudonymized sample-level ELISA and HI results, initial and repeat values, final classifications, and inclusion flags.
